# Nonlinear terahertz control of the lead halide perovskite lattice

**DOI:** 10.1126/sciadv.adg3856

**Published:** 2023-05-24

**Authors:** Maximilian Frenzel, Marie Cherasse, Joanna M. Urban, Feifan Wang, Bo Xiang, Leona Nest, Lucas Huber, Luca Perfetti, Martin Wolf, Tobias Kampfrath, X.-Y. Zhu, Sebastian F. Maehrlein

**Affiliations:** ^1^ Fritz Haber Institute of the Max Planck Society, Department of Physical Chemistry, Berlin, Germany.; ^2^ LSI, CEA/DRF/IRAMIS, CNRS, Ecole Polytechnique, Institut Polytechnique de Paris, Palaiseau, France.; ^3^ Department of Chemistry, Columbia University, New York City, NY, USA.; ^4^ Freie Universität Berlin, Berlin, Germany.

## Abstract

Lead halide perovskites (LHPs) have emerged as an excellent class of semiconductors for next-generation solar cells and optoelectronic devices. Tailoring physical properties by fine-tuning the lattice structures has been explored in these materials by chemical composition or morphology. Nevertheless, its dynamic counterpart, phonon-driven ultrafast material control, as contemporarily harnessed for oxide perovskites, has not yet been established. Here, we use intense THz electric fields to obtain direct lattice control via nonlinear excitation of coherent octahedral twist modes in hybrid CH_3_NH_3_PbBr_3_ and all-inorganic CsPbBr_3_ perovskites. These Raman-active phonons at 0.9 to 1.3 THz are found to govern the ultrafast THz-induced Kerr effect in the low-temperature orthorhombic phase and thus dominate the phonon-modulated polarizability with potential implications for dynamic charge carrier screening beyond the Fröhlich polaron. Our work opens the door to selective control of LHP’s vibrational degrees of freedom governing phase transitions and dynamic disorder.

## Teaser

Nonlinear THz excitation establishes coherent control over the octahedral twist modes of lead halide perovskites.

## INTRODUCTION

During the past decade, lead halide perovskites (LHPs) emerged as promising semiconductors for efficient solar cells, light-emitting diodes, and other optoelectronic devices (*1*). Key prerequisites for the high LHP device efficiencies are the long charge carrier diffusion lengths and lifetimes (*2*), often explained by the unusual defect physics (*3*) and/or dynamic charge carrier screening (*4*, *5*). The latter relies on delicate electron-phonon coupling, established by the dominant role of the static structure and dynamics of the lead-halide framework (*6*, *7*). However, the exact mechanisms of the carrier-lattice interaction in the highly polarizable and anharmonic LHP lattices remain debated (*8*, *9*). The sensitivity of the physical properties to structural distortions is a common feature in the extensive family of perovskites. In particular, for oxide perovskites, the control of specific lattice modes leads to ultrafast material control and nonlinear phononics (*10*, *11*). Successful examples include, among others, light-induced superconductivity (*12*), magnetization switching (*13*), access to hidden quasi-equilibrium spin states (*14*), ferroelectricity (*15*), and insulator-metal transitions (*16*) in perovskite or similar garnet structures.

**Fig. 1.**
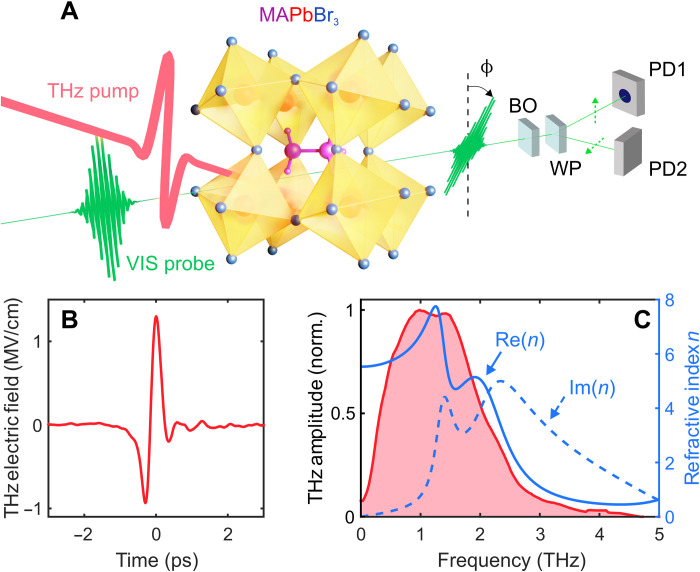
THz fields for nonlinear lattice control in LHPs. (**A**) Sketch of the experimental pump-probe configuration. An intense THz electric field causes a transient change of birefringence, leading to an altered probe pulse polarization. This change in polarization is read out using a balanced detection scheme, consisting of balancing optics (BO), Wollaston prism (WP), and two photodiodes (PD1 and PD2). (**B**) Used pump THz electric field measured using electro-optic sampling. (**C**) Complex refractive index of MAPbBr_3_ (blue curves) obtained from (*6*) and Fourier transform of the THz field (red area) in (B).

The crystal structure of LHPs features a large A-site cation surrounded by PbX_6_ octahedra consisting of lead (Pb) and halide (X) ions in the ABX_3_ crystal structure (see Fig. 1A). The electronic band structure is mainly determined by the identities of metal and halide but is also highly sensitive to the Pb-X-Pb bond angle, which can be controlled through the steric hindrance of the A-cation (*17*). Changing the Pb-X-Pb bond angle is equivalent to tilting of the PbX_6_ octahedra, which serves as an order parameter for the cubic ➔ tetragonal ➔ orthorhombic phase transitions (*18*, *19*). Octahedral tilting is also an important factor governing structural stability (*20*), dynamic disorder (*21*, *22*), and potential ferroelectricity (*5*, *23*) in LHPs. A recent study using resonant excitation of the ~1-THz octahedral twist mode (Pb-I-Pb bending) revealed modulation of the bandgap of CH_3_NH_3_PbI_3_ at room temperature (*24*). A similar observation of dynamic bandgap modulation due to twist modes was made at 80 K for off-resonant impulsive Raman excitation (*25*). These findings indicate an intriguing role of carrier coupling to Raman-active nonpolar phonons in addition to the polar longitudinal optical (LO) phonons in the conventional Fröhlich polaron picture (*7*, *26*). Moreover, the application of the Fröhlich polaron picture to LHPs has been questioned (*5*) because of the limited applicability of the harmonic approximation in these soft lattices (*8*).

Accordingly, the dynamic screening picture in LHPs is incomplete, and its microscopic mechanism continues to be debated (*27*, *28*). Furthermore, identifying and characterizing polaronic behavior is experimentally difficult (*26*, *28*–*32*). Optical Kerr effect (OKE) in LHPs (*33*, *34*) did not succeed in unveiling a lattice response and can be explained by an instantaneous electronic polarization (due to hyperpolarizability) instead (*35*). Moreover, previous strong field THz excitation could not directly detect the driven vibrational modes (*24*) and coherent control of the phonons remained elusive. Here, we turn to the THz-induced Kerr effect (TKE) (*36*, *37*) to investigate lattice-modulated polarization dynamics in the electronic ground state. We use intense THz electric fields (Fig. 1B) that broadly cover most of the inorganic cage modes (Fig. 1C) and may nonlinearly probe the THz polarizability. The rapidly changing single-cycle THz field macroscopically mimics the subpicosecond variation of local electric fields following electron-hole separation (*38*) and elucidates the isolated lattice response.

In general, the polarizability describes the tendency of matter to form an electric dipole moment when subject to an electric field, such as the local field from a mobile charge carrier in a semiconductor. In the presence of an electric field **E**, the microscopic dipole moment is given by **p**(**E**) = **μ**_0_ + α**E**, where **μ**_0_ is the static dipole moment and α is the polarizability tensor. In LHPs, α originates from three contributions: instantaneous electronic response (α_e_), lattice distortion (α_lat_), and molecular A cation reorientation (α_mol_). For small perturbations of the respective collective coordinate *Q* (charge distribution, molecular orientation, or lattice mode), a Taylor expansion yields

$$p(E,Q)=\mu_{0}+\frac{\partial \mu_{0}}{\partial Q}Q+\frac{\partial \alpha }{\partial Q}QE$$ (1)

where the two partial derivatives correspond to the mode effective charge Z^*^ and the Raman *R_ij_* tensor, respectively. Macroscopically, the two terms lead to lattice polarization Z^*^Q_IR_ and phonon-modulated susceptibility $\chi_{\mathrm{eq}}^{\left(1\right)}+[{\partial \chi }_{\mathrm{eq}}^{\left(1\right)}/\partial Q_{R}]Q_{R}$ for polar, *Q*_IR_, and nonpolar, *Q*_R_, modes, respectively. The latter relates ∂α/*∂Q* to a transient dielectric function and change in refractive index of the material. This relation thus enables studying microscopic polarizability through the observation of macroscopic transient birefringence induced by a pump pulse and experienced by a weak probe pulse (*36*, *37*). Collective polarization dynamics are induced by the driving force *F* = −*∂W*_int_/*∂Q*, where *W*_int_ = −**P**(**E**, *Q*) · **E** is the potential energy of the macroscopic polarization **P***=* ∑*_i_***p***_i_* interacting with an electric field **E **(from a local charge carrier or through light-matter coupling in the electric dipole approximation). Thus, two *E*_THz_ interactions lead to THz polarizability-induced transient birefringence in TKE (*37*), which is linearly probed by a weak probe pulse *E*_pr_ in an effective third-order nonlinear process proportional to χ^(3)^*E*_THz_*E*_THz_*E*_pr_ (see Materials and Methods) (*36*, *39*).

## RESULTS

### Experiment

To induce polarization dynamics, we use intense THz single-cycle pulses with a 1.0-THz center frequency (>1.5-THz spectral width; see Fig. 1C), delivering subpicosecond peak electric fields exceeding 1.5 MV/cm generated by optical rectification in LiNbO_3_ (*40*). We probe the resulting transient birefringence, i.e., anisotropic four-wave mixing (FWM) signals, by stroboscopic sampling with a synchronized 20-fs pulse (800-nm center wavelength) in a balanced detection scheme (see Fig. 1A). We therefore effectively measure a third-order nonlinear signal field heterodyned with the transmitted probe field. The probe pulses are linearly polarized at 45° with respect to the vertically polarized THz pulses. As representative LHPs, we investigate hybrid organic-inorganic CH_3_NH_3_PbBr_3_ (MAPbBr_3_) and fully inorganic CsPbBr_3_. The freestanding single-crystal samples (200- to 500-μm thickness) were solution-grown by an antisolvent diffusion method (see Materials and Methods) (*41*, *42*). Complementary polycrystalline thin films (~400-nm thickness) were spin-coated on 500-μm-thick BK7 substrates, being particularly technologically relevant as most state-of-the-art LHP solar cells are fabricated in a similar way (*1*).

### Room temperature TKE

**Fig. 2.**
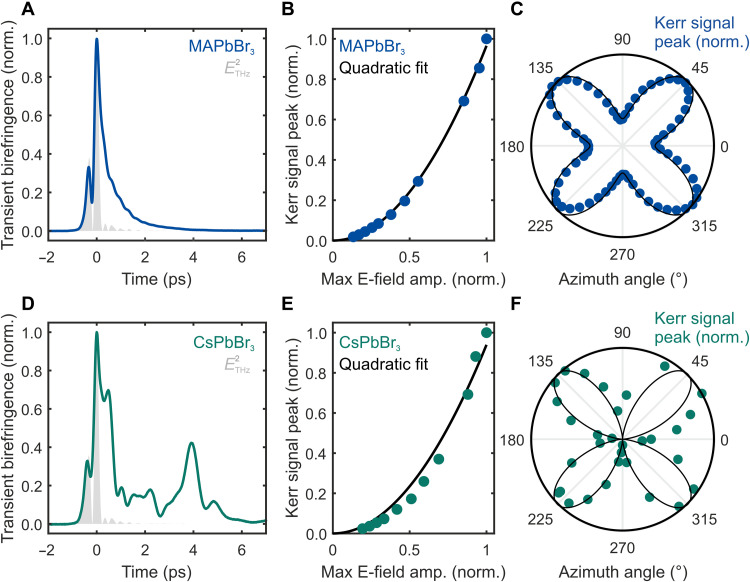
THz-induced birefringence in MAPbBr_3_ and CsPbBr_3_ at room temperature. (**A**) Room temperature TKE in MAPbBr_3_ and (**D**) CsPbBr_3_ single crystals. (**B** and **E**) THz fluence dependence of the transient birefringence peak amplitude with quadratic fit (black line), demonstrating the Kerr effect nature of the signals. (**C** and **F**) Azimuth angle (between probe beam polarization and crystal facets) dependence of main TKE peak with fit (black line) to expected χ^(3)^ tensor geometries in cubic and orthorhombic phase, respectively.

Figure 2 shows the THz-induced transient birefringence in MAPbBr_3_ single crystals at room temperature. The signal (blue line) initially follows $E_{\mathrm{THz}}^{2}$ (gray area, measured via electrooptic sampling) but then transitions into a nearly monoexponential decay for time delays *t* > 500 fs. The transient birefringence peak at *t* = 0 clearly scales quadratically with the THz field amplitude as found by the pump fluence dependence in Fig. 2B. With the exponential decay dynamics remaining also constant for different fluences (fig. S4), we can infer the Kerr-type origin of the full signal and thus conclude a strong THz polarizability. Furthermore, the peak amplitude’s (Fig. 2C) and the exponential tail’s (fig. S5) dependence on the azimuthal angle between probe polarization direction and crystal axes perfectly obeys the expected fourfold rotational symmetry of the χ^(3)^ tensor and TKE dependence of $\chi_{ijkl}^{\left(3\right)}E_{j}^{\mathrm{THz}}E_{k}^{\mathrm{THz}}E_{l}^{\mathrm{pr}}$. We quantify the THz polarizability of MAPbBr_3_ by a nonlinear THz refractive index *n*_2_ of about 2 × 10^−14^ cm^2^/W (see details in the Supplementary Materials), being on the same order as in the optical region (*43*) and roughly 80 times larger than *n*_2_ of diamond (*44*), which is known as a suitable material for THz nonlinear optics (*45*).

The small oscillatory deviations from the exponential tail in MAPbBr_3_ (Fig. 2A) become more pronounced and qualitatively different in CsPbBr_3_ in the form of a bumpy, nontrivial shape (Fig. 2D). This stark difference between MAPbBr_3_ and CsPbBr_3_ is reminiscent of two-dimensional (2D)–OKE results (*35*), where the oscillatory signal of CsPbBr_3_ was found to be mainly due to anisotropic light propagation, since CsPbBr_3_ is orthorhombic and thus birefringent at room temperature. The fluence (Fig. 2E) and azimuthal dependences (Fig. 2F) are consistent with the pure third-order nonlinearity of the signal. However, fits to the azimuthal angle dependences in Fig. 2 (C and F, black lines) yield different ratios of the diagonal $\chi_{iiii}^{\left(3\right)}$ to off-diagonal $\chi_{ijkl}^{\left(3\right)}$ tensor elements for the two materials: 1.6 for MAPbBr_3_ and 1.0 for CsPbBr_3_. A similar polarization dependence of static Raman spectra was recently attributed to additional isotropic disorder from the rotational freedom of the polar MA^+^ cation in MAPbBr_3_ (*46*).

### Temperature dependence: Single crystal versus thin film

**Fig. 3.**
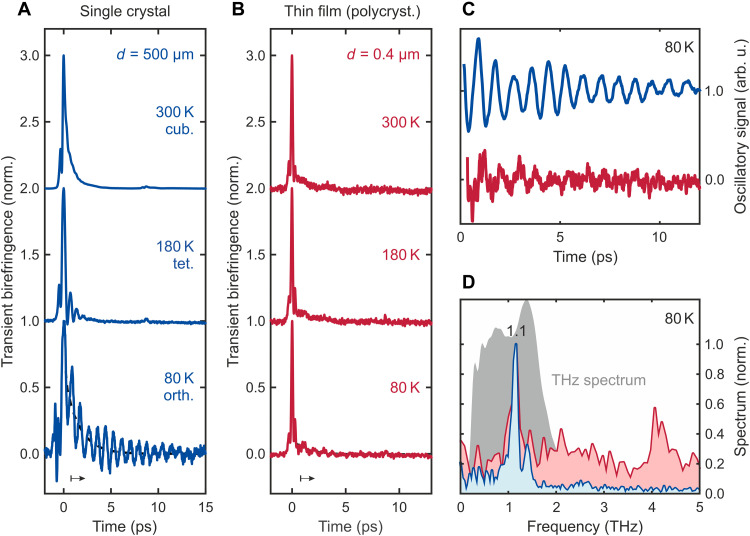
TKE temperature dependence of single-crystal versus thin-film MAPbBr_3_. (**A**) Temperature-dependent TKE for single-crystal and (**B**) thin-film samples. (**C**) Oscillatory signal components at 80 K extracted by subtracting the non-oscillatory tail (dashed lines) and starting after the main peak (bottom black arrow) in (A) and (B). (**D**) Respective Fourier transforms (blue and red) of (C) and incident THz pump spectrum (gray area).

Figure 3 (A and B) shows a comparison of the temperature-dependent TKE in MAPbBr_3_ single crystals and polycrystalline thin films. At room temperature (both top traces), it stands out that the thin-film TKE signal lacks the exponential decay seen in the single crystals, providing a first evidence that the tail stems from dispersion effects and is not due to intrinsic molecular relaxation dynamics as previously interpreted (*47*). A strong THz dispersion, as seen in Fig. 1C, is a general, but often overlooked, phenomenon in broadband high-field THz pump-probe spectroscopy. In analogy to the OKE (*35*), the features of the room temperature TKE in both MAPbBr_3_ and CsPbBr_3_ might therefore be dominated by dispersive and anisotropic light propagation. Hence, we assign the main contribution of the TKE response at room temperature to the instantaneous electronic polarizability (hyperpolarizability), which may overwhelm possible lattice contributions. This interpretation will be further supported by the modeling below.

From here on, we mainly focus on the TKE of MAPbBr_3_, especially at low temperatures at which increased phonon lifetimes should facilitate the observation of a coherent lattice response (*46*). For the single crystal (Fig. 3A), the TKE dynamics at 180 K are different than at room temperature, which might reflect the change of structural phase from cubic to tetragonal. At 180 K, an oscillatory signal at short times (<2 ps) appears, suggesting the presence of a coherent phonon, which was overdamped in the cubic phase at room temperature (*46*). The oscillations become much stronger for the single crystal at 80 K, where MAPbBr_3_ is in the orthorhombic phase. Less pronounced but clear oscillations are also visible in the thin-film sample at 80 K (Fig. 3A, lowest trace). We extract the oscillatory parts (Fig. 3C) of both single-crystal and thin-film samples at 80 K by subtracting incoherent backgrounds (see the Supplementary Materials and figs. S11 and S12 for details) using a convolution of the squared THz field with a temporal delta function and biexponential function. The respective Fourier transforms in Fig. 3D reveal the same oscillations frequency of 1.15 ± 0.05 THz for both samples. This clearly rules out anisotropic propagation effects as the origin of these oscillations (*35*), because the 400-nm film is too thin for a walk-off between pump and probe (shown in simulations later) and the different thicknesses of the two samples rule out a Fabry-Pérot resonance effect. The broader 4.2-THz thin-film feature is constrained to a short time window of 0.5 < *t* < 2 ps (see fig. S13) and potentially related to thin-film substrate interference effects. Thus, we can overall assign the main oscillatory signal to a lattice modulation of the THz polarizability dominated by a single 1.15-THz phonon in MAPbBr_3_. We now turn to THz-THz-Visible FWM simulations to understand the origins of TKE from MAPbBr_3_.

### Modeling

For dispersive and birefringent materials, the Kerr signal cannot be decomposed into an effective birefringence change observed by an independent probe beam (*39*). Instead, the Kerr effect–induced nonlinear polarization **P**^(3)^ needs to be captured in a full FWM picture. To separate the three polarizability contributions (instantaneous electronic, molecular, and lattice) and to take anisotropic light propagation across dispersive phonon resonances into account, we simulate the third-order nonlinear polarization by

$$P_{i}^{\left(3\right)}(t,z)=\epsilon_{0}\int_{-\infty }^{t}{dt}^{\prime }\int_{-\infty }^{t^{\prime }}dt^{\prime \prime }\int_{-\infty }^{t^{\prime \prime }}dt^{\prime \prime \prime }{\tilde{R}}_{ijkl}R(t,t^{\prime },t^{\prime \prime },t^{\prime \prime \prime })E_{j}^{\mathrm{THz}}(t^{\prime },z)E_{k}^{\mathrm{THz}}(t^{\prime \prime },z)E_{l}^{\mathrm{pr}}(t^{\prime \prime \prime },z)$$ (2)

where *R* is the time-domain χ^(3)^ response function (*39*) and **E**^THz^ and **E**^pr^ are the pump and probe electric fields, respectively. The time-independent ${\tilde{R}}_{ijkl}$ tensor constitutes the respective χ^(3)^ symmetry for the different crystalline phases, in agreement with the ratios of the tensor elements obtained from the azimuthal fits in Fig. 2C. For the instantaneous electronic polarizability (hyperpolarizability), we assume temporal Dirac delta functions *R*_e_(*t*, *t*′, *t*′′, *t*′′′) = *R*_e,0_δ(*t* − *t*′)δ(*t*′ − *t*′′)δ(*t*′′ − *t*′′′). For a lattice response, we model the driven phonon response by a Lorentz oscillator

$$\begin{array}{r} R_{\mathrm{ph}}(t,t^{\prime },t^{\prime \prime },t^{\prime \prime \prime })=R_{\mathrm{ph},0}\delta (t^{\prime }-t^{\prime \prime })\delta (t^{\prime \prime }-t^{\prime \prime \prime })e^{-\text{Γ}(t-t^{\prime })}\sin\left[\sqrt{\left(\omega_{\mathrm{ph}}^{2}-{\text{Γ}}^{2}\right)}(t-t^{\prime })\right] \end{array}$$ (3)

where ω_ph_/2π is the frequency and 1/2Γ is the lifetime of the phonon (*39*). The driving force for Raman-active phonons is hereby $E_{j}^{\mathrm{THz}}E_{k}^{\mathrm{THz}}$, which contains difference-frequency (DF) and sum-frequency (SF) terms (*48*, *49*). The latter is a unique distinction to the OKE. For **E**^THz^, we can directly use the experimental THz electric field, as measured in amplitude and phase-resolved electro-optic sampling. After we determine the complex refractive indices (Fig. 1C) and extrapolate the static birefringence (see Materials and Methods and the Supplementary Materials), we calculate and propagate all involved fields from Eq. 2, including signal fields $E_{i}^{\left(4\right)}(t,z)$ emitted from $P_{i}^{\left(3\right)}(t,z)$, followed by our full detection scheme, including balanced detection, to obtain the pump-probe signal (see details in Materials and Methods).

**Fig. 4.**
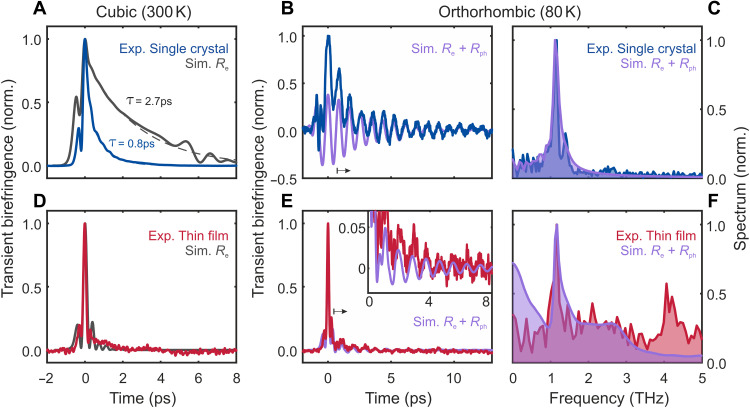
FWM simulations versus experimental results in MAPbBr_3_. Isotropic cubic phase (300 K): Simulated TKE signals for (**A**) single crystal (500-μm thickness) and (**D**) thin film (400-nm thickness) assuming only instantaneous electronic response *R*_e_(*t*) (gray lines). Anisotropic orthorhombic phase (80 K): (**B**) Single-crystal and (**E**) thin-film TKE versus simulation for model system with static birefringence, instantaneous electronic *R*_e_(*t*) and Lorentz oscillator *R*_ph_(*t*) phonon response (purple lines). (**C** and **F**) Fourier transforms of experimental data (blue and red) and simulation results (purple) from (B) and (E), respectively, normalized to the phonon amplitude at 1.15 THz.

Figure 4A shows the simulated TKE signal (gray) compared to the experimental data (blue) at room temperature for a 500-μm-thick MAPbBr_3_ single crystal. It unveils the formation of a long exponential tail produced by walk-off, dispersion, and absorption effects, even for only an instantaneous electronic response *R*_e_. This confirms that the electronic polarizability dominates the TKE signal at room temperature. It contrasts a previous interpretation of a TKE measurement in thick single-crystal MAPbBr_3_, which neglected propagation effects entirely (*47*). At 80 K, MAPbBr_3_ is orthorhombic. We therefore need to include additional static birefringence. Instantaneous hyperpolarizability alongside static birefringence and dispersion can cause the appearance of oscillatory features (*35*). Nevertheless, our modeling finds these features to be too short-lived (see fig. S17) to explain our experimental observation at 80 K. The absence of oscillatory propagation effects in MAPbBr_3_’s experimental TKE can be explained by the nearly vanishing instantaneous hyperpolarizability contribution at 80 K in Fig. 3A. Nevertheless, we account for both hyperpolarizability *R*_e_ and lattice-modulated polarizability *R*_ph_ responses [fit parameters: ω_ph_/2π = 1.14 THz, Γ = (2 · 1.7 ps)^−1^, *R*_e,0_/*R*_ph,0_ = 2.4] to describe the low-temperature TKE signals in the time and frequency domains (Fig. 4, B and C). In contrast to OKE at 80 K (*35*), the oscillations in TKE are therefore due to coherent phonon modes, and we hence finally observe an ultrafast lattice response to a subpicosecond electric field transient.

**Fig. 5.**
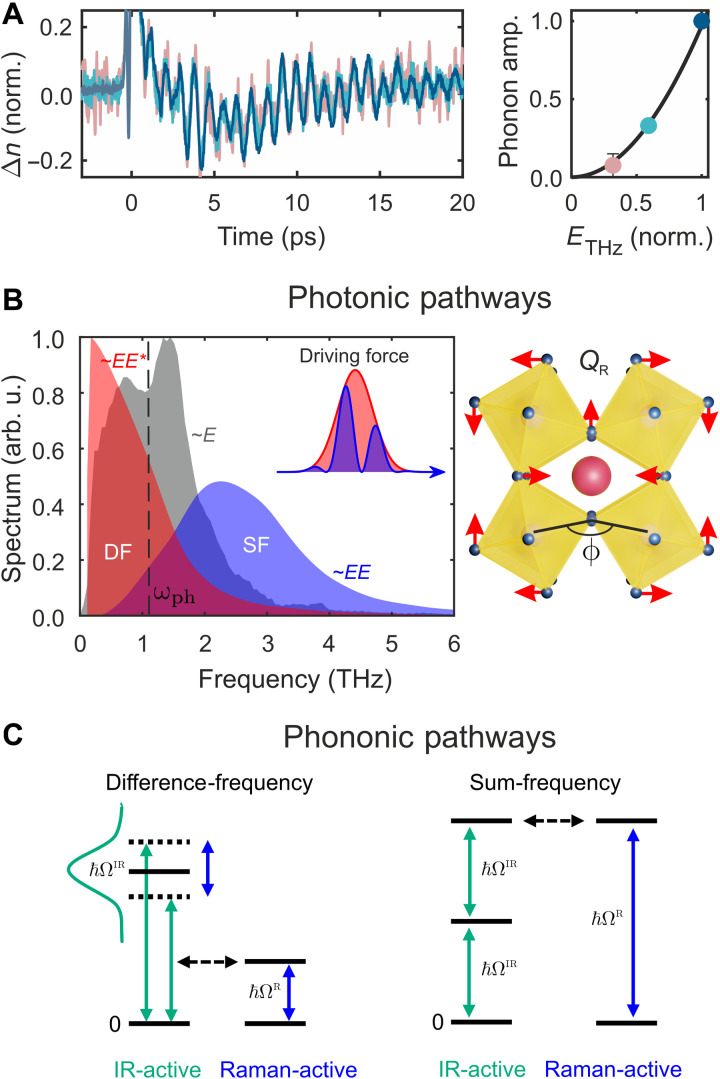
Nonlinear excitation pathways for the 1.15-THz Raman-active twist mode. (**A**) Time domain coherent phonon oscillations (normalized to *t* = 0 TKE main peak) in MAPbBr_3_ single crystal at 80 K for different THz field strengths (left) and respective coherent phonon amplitude obtained from Fourier transform (right), both unveiling an $E_{\mathrm{THz}}^{2}$ scaling law and thus demonstrating a nonlinear excitation. (**B**) Possible nonlinear photonic excitation pathways for the ω_ph_ = 1.15 THz mode (dashed line) mediated via a THz electronic polarizability. The nonlinearly coupled *E*_THz_ spectrum (gray area) leads to difference-frequency $E_{\mathrm{THz}}E_{\mathrm{THz}}^{*}$ (DF; red area) and sum-frequency *E*_THz_*E*_THz_ (SF; blue area) driving forces. The octahedral twist mode is schematically sketched on the right-hand side. (**C**) Possible phononic pathways via a directly driven IR-active phonon *Q*_IR_, which nonlinearly couples to the Raman-active mode *Q_R_* via anharmonic $Q_{R}Q_{\mathrm{IR}}^{2}$ coupling.

The simulation assuming only instantaneous hyperpolarizability for a 400-nm-thin film agrees well with the experimental TKE at room temperature (see Fig. 4D). As expected, the simulation lacks the clear tail seen in the thick single crystals, thereby additionally confirming that the tail is due to light propagation effects. Also here, at 80 K, we need to include both instantaneous electronic and phonon contributions [ω_ph_/2π = 1.14 THz, Γ = (2 · 1.7 ps)^−1^, *R*_e,0_/*R*_ph,0_ = 24] to describe the experimental signals for the thin films in Fig. 4 (E and F). We do not include the 4.2-THz feature into our model due its peculiar temporal origin limited to the first 2 ps (see fig. S13). The broad shoulder centered at 2.5 THz, nevertheless, is well captured by our model as it is given by the SF components of the instantaneous electronic response (see Fig. 5B, blue area). Notably, the simulation confirms that a purely instantaneous electronic contribution alongside static birefringence does not lead to oscillatory features in thin films (see fig. S17, A and C). This provides direct proof that the observed 1.15-THz oscillations in Fig. 3 (C and D) originate from a coherent phonon. The ratio of the instantaneous peak versus coherent phonon oscillation amplitude is noticeably different between the single crystal and thin film. This may be due to the larger low-frequency (see fig. S14B) penetration depth, which favors the phonon’s SF excitation (see details later), and the dispersion effects, which smear out the instantaneous peak in the thick single crystal. Through comparison of single crystals with thin films and by rigorous FWM simulation, we have therefore proven to witness a coherent lattice-driven dynamic polarization response.

### Interpretation

Besides potential rotational disorder, our rigorous modeling shows that we do not observe a TKE contribution that we can unambiguously relate to an ultrafast cation reorientation in the form of a liquid-like exponential decay (*36*, *37*). We rather find MAPbBr_3_’s TKE tail at room temperature to be most likely overwhelmed by the instantaneous hyperpolarizability *R*_e_ in conjunction with dispersive light propagation. This might be also explained by the THz pump spectrum being far off the cation rotational resonances around the 100-GHz frequency range (*1*). The cation species nevertheless influences the static and dynamic properties of the inorganic lattice, highlighting the importance of the interplay between the organic and inorganic sublattices for the LHP equilibrium structure (*46*). This fact shows up, e.g., as a single dominating PbBr_6_ cage mode in MAPbBr_3_ but two dominating modes in CsPbBr_3_ (see fig. S1), in agreement with static Raman spectra (*46*). The various templating mechanisms by which the cation influences these properties include its steric size (*17*) and lone-pair effects (*22*, *23*).

For MAPbBr_3_, we find a single phonon mode dominating the Raman-active lattice dynamics in response to a subpicosecond electric field spike. The observed phonon at 1.15 THz is consistent with static Raman spectra in the visible range, where this mode also exhibits the highest scattering amplitude (*46*, *50*). Thus, we can assign it to a dynamic change in the Pb-Br-Pb bond angle corresponding to a twisting of the PbBr_6_ octahedra (twist mode) (*51*). On the basis of theory work for MAPbI_3_ (*52*), we assign this to A_g_ symmetry, which matches the experimental observations that the mode is still present when we rotate the single crystal by 45° (see fig. S9) and that we also observe the same mode in polycrystalline thin films (Fig. 3, C and D). We suggest that at room temperature, this mode also strongly modulates the THz dielectric response, although its oscillations are potentially overdamped, as inferred from the broad Raman spectra (*46*). To distinguish whether this twist mode only dominates the ultrafast lattice response in MAPbBr_3_, or is of wider relevance for other LHPs, we analyze the TKE response of orthorhombic phase CsPbBr_3_ (see fig. S1), where we observe two dominating modes at 0.9 and 1.3 THz at 80 K, corresponding to two octahedra twist modes. Smaller features around 2 THz indicate additional contributions from Pb-Br stretch modes, also consistent with static Raman spectra (*46*). Studies of the high-temperature cubic phase CsPbBr_3_ at *T* > 403 K remained challenging because of CsPbBr_3_’s temperature instability and are therefore left to future studies. From the MAPbBr_3_ versus CsPbBr_3_ comparison, we can conclude that the transient THz polarizability $\left[\partial \chi_{\mathrm{eq}}^{\left(1\right)}/\partial Q\right]Q$ is generally dominated by the octahedra twist modes in LHPs.

We now consider the excitation mechanism of the coherent phonon. Figure 5A shows that the 1.15-THz oscillations at 80 K scale with the square of the THz electric field amplitude, suggesting nonlinear excitation with a Raman-type driving force. This is consistent with the Kerr effect being also a Raman-type probing mechanism. In general, there are four types of Raman-active THz excitation mechanisms: DF or SF excitation via ionic Raman scattering (IRS) or stimulated Raman scattering, corresponding to nonlinear ionic (= phononic) or nonlinear electronic (= photonic) pathways, respectively (*48*, *49*). The A_g_ symmetry of the observed modes permits IRS, where a resonantly driven infrared (IR)–active phonon couples anharmonically to a Raman-active mode (*10*, *49*). However, this phononic pathway requires phonon anharmonicity, whereas the photonic pathway requires electronic THz polarizability. The SF and DF photonic force spectra in Fig. 5B indicate a comparable probability for both photonic mechanisms to drive the 1.15-THz mode (dashed line). For the phononic pathways in Fig. 5C, the DF excitation requires a primarily driven IR-active phonon with a bandwidth of ≳1 THz, which exists in our excitation range even at 80 K (*53*). On the other hand, there are also IR-active modes, which provide roughly half the frequency of the Raman-active mode Ω_IR_ = Ω_R_/2 enabling phononic SF-IRS (*49*). Accordingly, none of the four nonlinear excitation pathways can be neglected, but the observed strong electronic THz polarizability in conjunction with a longer penetration depth for lower THz frequencies favors an SF nonlinear photonic mechanism. We leave the determination of the exact excitation pathway to further studies, e.g., by 2D THz spectroscopy (*54*) or more narrowband THz excitation (*55*).

## DISCUSSION

Independent of the precise excitation pathway and in contrast to optical Raman or transient absorption studies, we unambiguously observe strong electron-phonon coupling of the octahedral twist modes via a pure THz polarizability (electronic or ionic). This explains the mode’s dominating influence on the electronic bandgap in MAPbI_3_ previously observed by Kim *et al.* (*24*). The twist mode’s half-cycle period of ~0.5 ps is short enough to contribute to electron-phonon coupling within the estimated polaron formation time (*56*), even in the overdamped case at room temperature. We can understand carrier screening by nonpolar modes as follows. As shown in Eq. 1, the THz polarizability contains two lattice contributions: from polar lattice modes *P*_IR_(ω) ∝ Z^*^Q_IR_(ω) ∝ Z^*^E_THz_(ω) and from the nonresonant electron cloud moving at THz speeds (subpicosecond time scales)

$$P_{e}(\omega )=\epsilon_{0}\left[\chi_{e}^{\left(1\right)}(\omega )+\frac{{\partial \chi }_{e}^{\left(1\right)}}{\partial Q_{R}}(\omega ,\text{Ω})\;Q_{R}(\text{Ω})\right]E_{\mathrm{THz}}(\omega )$$ (4)

where the latter is modulated in the presence of a Raman-active phonon *Q*_R_. Thus, excited Raman-active modes lead to a transient dielectric response ε(ω) = ε_eq_(ω) + Δε(ω, Ω) at THz frequencies ω with $\text{Δ}\epsilon =\frac{{\partial \chi }_{e}^{\left(1\right)}}{\partial Q_{R}}Q_{R}$, which constitutes an additional contribution of higher-order screening due to a fluctuating lattice. In the macroscopic incoherent case, Δε averages out. On time and length scales relevant to electron-hole separation and localization (<1 nm and <1 ps) (*38*), collective octahedral tilting produces (*57*) an additional THz polarizability, which might add to the conventional Fröhlich picture of carrier screening. We speculate that a local nonequilibrium twist angle *Q*_R_ either could be already present because of dynamic disorder (see discussion below) (*57*) or might be nonlinearly excited by the transient local charge field $E_{\mathrm{loc}}^{2}$, easily exceeding 1 MV/cm (analog to the excitation pathways above) (*4*). The latter scenario agrees with MAPbBr_3_’s unusually large optical χ^(3)^, previously attributed to local confinement effects (*43*). The observed 1.15-THz mode is therefore a good candidate for contributing to strong electron-phonon coupling beyond the polar Fröhlich picture.

The driven twist mode is similar to soft modes in oxide perovskites, where the tilting angle of adjacent oxygen octahedra is an order parameter for phase transitions (*58*). Recently, TKE was similarly used to drive and detect ultrafast field-induced ferroelectricity in the quantum paramagnet SrTiO_3_ (*15*). In Eu- and Sr-doped La_2_CuO_4_, driving the tilt of oxygen octahedra was found to induce signatures of superconductivity persisting over a few picoseconds above the critical temperature (*12*). Consistent with these observations in oxide perovskites, the tilting angle of the PbX_6_ octahedra (twist mode) was found to act as an order parameter for phase transitions in LHPs (*18*, *19*). Especially for MAPbBr_3_, the Raman scattering intensity of the 1.1-THz peak was recently shown as measure of the orthorhombic-tetragonal phase transition (*50*) and its spectral evolution in Raman (*46*) and neutron scattering (*59*) is indicative of a soft mode phase transition. However, the LHP lattice properties were previously mainly tuned in a static and chemical manner, e.g., by acting on the octahedral tilting angle through the steric size of the A-site cation (*17*). The coherent lattice control demonstrated here allows dynamic tuning of the structure (twisting angles estimated in the Supplementary Materials) and thus ultrafast phonon-driven steering of LHP’s optoelectronic properties, e.g., for integrated photonic devices operating at GHz to THz clock rates (*60*). Using this knowledge for chemical engineering, experts may try to design structures with specific static octahedral distortions and enhanced twist dynamics, e.g., by substituting the metal cation to create materials stable or unstable with respect to octahedral rotation (*61*).

In addition, imposing a coherence on the octahedral tilting should directly influence the dynamic disorder (*61*), which is considered one of the key components determining the optoelectronic properties of LHPs (*8*, *32*, *46*). Dynamic disorder means that the effective crystallographic structure (e.g., cubic at 300 K) only exists in spatial and temporal average. Specifically, in LHPs with a Goldschmidt tolerance factor below 1, such as MAPbBr_3_ and CsPbBr_3_, the disorder mainly arises from the lattice instability associated with octahedral tilting (*21*, *22*, *61*), evidenced by inelastic x-ray scattering in MAPbI_3_ (*57*) and Raman spectroscopy in MAPbBr_3_, CsPbBr_3_, and MAPbI_3_ (*21*, *46*). The resulting fluctuating lattice potential and polar nanodomains have been suggested as underlying mechanisms for dynamic charge carrier screening in the form of preferred current pathways (*62*) and ferroelectric polarons (*5*), respectively. All these phenomena might be potentially controlled or temporally lifted by the THz control of octahedral motion.

Overall, we find that the octahedral tilting motion, which serves as an order parameter for phase transitions (*18*, *19*) and contributes substantially to dynamic disorder (*21*, *46*), shows a strong nonlinear coupling to a rapidly varying electric field on subpicosecond time scales that are relevant to local electron-hole separation and polaron formation. Our results thus indicate that the transverse optical octahedral twist mode contributes to strong electron-phonon coupling and dynamic carrier screening in LHPs, which may be inherently linked to a local and transient phase instability as suggested by the ferroelectric polaron picture (*5*).

In conclusion, by investigating third-order nonlinear polarization dynamics in hybrid and all-inorganic LHPs, we reveal that the room temperature TKE response stems predominantly from a strong THz hyperpolarizability, leading to a nonlinear THz refractive index on the order of 10^−14^ cm^2^/W. In analogy to previous OKE studies (*35*), we explain and model the appearance of retarded TKE dynamics by dispersion, absorption, walk-off, and anisotropy effects (*39*). These effects are of crucial relevance to contemporary THz pump-probe experiments. For sufficiently long phonon lifetimes at lower temperatures, we can nonlinearly drive and observe a coherent lattice response of the ~1-THz octahedral twist mode(s). These phonons couple most strongly to the THz polarizability, which means that they must be highly susceptible to transient local fields on the hundreds of femtoseconds time scale, relevant to electron-phonon coupling and carrier localization. We find this ultrafast nonpolar lattice response to be mediated by anharmonic phonon-phonon coupling and/or by the strong nonlinear electronic THz polarizability. The same octahedral twist mode serving as a sensitive order parameter for structural phase transitions (*50*, *59*) is likely the origin of substantial intrinsic dynamic disorder in LHPs (*46*, *61*). Thus, our findings suggest that the microscopic mechanism of the unique defect tolerance (*34*, *63*) and long carrier diffusion lengths (*1*, *2*) of LHPs might also rely on small phase instabilities accompanying the polaronic effects.

Our work demonstrates the possibility of coherent control over the twist modes via nonlinear THz excitation. Since the octahedral twist modes are the dynamic counterparts to steric engineering of the metal-halide-metal bond angle, our work paves the way to study charge carriers in defined modulated lattice potentials, to control dynamic lattice disorder, or to macroscopically switch polar nanodomains, leading to the emergence of transient ferroelectricity.

## MATERIALS AND METHODS

### Sample growth

The single-crystal samples were synthesized on the basis of our previously published method (*35*). For MAPbBr_3_, the precursor solution (0.45 M) was prepared by dissolving equal molar ratio of MABr (Dyesol, 98%) and PbBr_2_ (Sigma-Aldrich, ≥98%) in dimethylformamide (DMF; Sigma-Aldrich, anhydrous 99.8%). After filtration, the crystal was allowed to grow using a mixture of dichloromethane (Sigma-Aldrich, ≥99.5%) and nitromethane (Sigma-Aldrich, ≥96%) as the antisolvent (*41*). Similar method was used for CsPbBr_3_ crystal growth (*42*). The precursor solution (0.38 M) was formed by dissolving equal molar ratio of CsBr (Sigma-Aldrich, 99.999%) and PbBr_2_ in dimethyl sulfoxide (DMSO; EMD Millipore Co., anhydrous ≥99.8%). The solution was titrated by methanol until yellow precipitates show up and did not redissolve after stirring at 50°C for a few hours. The yellow supernatant was filtered and used for the antisolvent growth. Methanol was used for the slow vapor diffusion. All solid reactants were dehydrated in a vacuum oven at 150°C overnight, and all solvents were used without further purification.

### Thin films

Before spin-coating, the substrate was rinsed by acetone, methanol, and isopropanol and treated under oxygen plasma for 10 min. The freshly prepared substrate was transferred to the spin coater within a short time. For MAPbBr_3_, the precursor DMSO (Sigma-Aldrich, ≥99.9%) solution (2 M) containing the equimolar ratio of MABr and PbBr_2_ was used for the one-step coating method. The film was formed by spin-coating at 2000 rpm for 45 s and annealed at 110°C for 10 min. For CsPbBr_3_, a two-step method was implemented. First, the PbBr_2_ layer was obtained by spin-coating the 1 M PbBr_2_/DMF precursor solution at 2000 rpm for 45 s and dried at 80°C for 30 min. Subsequently, the PbBr_2_ film was immersed in a 70 mM CsBr/methanol solution for 20 min. Following the rinsing by isopropanol, the film was annealed at 250°C for 5 min to form the uniform perovskite phase.

### THz-induced Kerr effect

THz pulses with 1.0-THz center frequency and field strength exceeding 1.5 MV/cm (Fig. 1, B and C) were generated by optical rectification in LiNbO_3_ with the tilted pulse front technique (*40*). To that end, LiNbO_3_ was driven by laser pulses from an amplified Ti:sapphire laser system (central wavelength, 800 nm; pulse duration, 35 fs full width at half maximum; pulse energy, 5 mJ; repetition rate, 1 kHz). The probe pulses came from a synchronized Ti:sapphire oscillator (center wavelength, 800 nm; repetition rate, 80 MHz) and were collinearly aligned and temporarily delayed with respect to the THz pulse. The probe polarization was set at 45° with respect to the vertically polarized THz pulses. The THz pulses induced a change in birefringence (TKE) in the sample (*36*). This birefringence causes the probe field to acquire a phase difference between polarization components parallel and perpendicular to THz pulse polarization. The phase difference is detected via a half- and quarter-wave plate followed by a Wollaston prism to spatially separate perpendicularly polarized probe beam components. The intensity of the two beams is detected by two photodiodes in a balanced detection configuration.

### FWM simulation

The third-order nonlinear polarization **P**^(3)^(*t*, *z*) is simulated using the general FWM equation (Eq. 2) and according to (*39*). To compute **P**^(3)^(*t*, *z*), all three contributing light fields, $E_{j}^{\mathrm{THz}},E_{k}^{\mathrm{THz}}$, and $E_{l}^{\mathrm{pr}}$, are propagated through the crystal on a time-space grid. The three fields inside the sample are calculated at any location *z* using

$$E_{i}(t,z)=\int_{-\infty }^{\infty }t_{i}(\omega )A_{i}(\omega )e^{-i[\omega t-k_{i}(\omega )z]}[1-R_{i}(\omega ,z)]d\omega$$ (5)

with

$$R_{i}(\omega ,z)=r_{i}[1+e^{2izk_{i}(\omega )}]\frac{e^{2i(d-z)k_{i}(\omega )}}{1-r_{i}^{2}(\omega )e^{2idk_{i}(\omega )}}$$ (6)

where *A_i_*(ω) is the spectral amplitude of the field, and *t_i_* and *r_i_* denote the Fresnel transmission and reflection coefficients, respectively. As the input pump field **E**^THz^, we use the full experimental THz electric field generated via optical rectification in LiNbO_3_ as measured using electro-optic sampling in quartz. For the probe field **E**^pr^, we assume a Fourier-limited Gaussian spectrum with a center wavelength of 800 nm and a pulse duration of 20 fs, experimentally measured by a spectrometer and a commercial SPIDER. For both non-birefringent and birefringent simulations, we use the THz refractive index for MAPbBr_3_, as calculated from its dielectric function based on the experimental work by Sendner *et al.* (*6*) (fig. S14). In the optical region, the precise anisotropic refractive index of CsPbBr_3_ is used as measured using the 2D-OKE (*39*). For the birefringent LHP simulation, the static birefringence of CsPbBr_3_ is used and interpolated to THz region (fig. S15). For the isotropic cubic perovskite, the static birefringence is set to zero. In the shown simulation results, the time grid had a finite element size of Δ*t*′ = 16.6 fs and the spatial grid had a finite element size of Δ*z* = 10 μm for the single-crystal and Δ*z* = 0.1 μm for the thin-film simulations, respectively. These values were chosen for the sake of computational efficiency and did not qualitatively affect the simulation results. The pump-probe delay finite element size was chosen to be Δ*t* = 16.6 fs.

We assume that the nonlinear polarization **P**^(3)^ emits an electric field **E**^(4)^ at every slice *z* according to the inhomogeneous wave equation

$$\left[\nabla^{2}+k_{i}^{2}(\omega )\right]E_{i}^{\left(4\right)}(\omega ,t,z)=-\frac{\omega^{2}}{\epsilon_{0}c^{2}}P_{i}^{\left(3\right)}(\omega ,t,z)$$ (7)

which then copropagates with the probe field **E**^pr^. The transmitted probe field **E**^pr^ and emitted field **E**^(4)^ are projected on two orthogonal polarization components by propagating through a half-wave plate, quarter-wave plate, and Wollaston prism. The combined effect of these optical devices is captured by the Jones matrices *J*_1_ and *J*_2_ for the two separated polarization component channels. A balanced detection scheme allows observation of **E**^(4)^ by interfering with **E**^pr^. Under balanced conditions, the detected nonequilibrium signal is

$$S\propto \int Re\{(J_{1}E^{\mathrm{pr}})\;\cdot [J_{1}E^{(4)*}]-(J_{2}E^{\mathrm{pr}})\;\cdot [J_{2}E^{(4)*}]\}d\omega$$ (8)

Our simulation therefore mimics the balancing conditions of the experiment. A detailed description of this calculation is given in (*39*).

To model the response of the system, we assume the response function *R*_e_(*t*, *t*′, *t*′′, *t*′′′) for an instantaneous electronic response and *R*_ph_(*t*, *t*′, *t*′′, *t*′′′) for a phonon response. The expressions for *R*_e_ and *R*_lat_ are given here. In the frequency domain, *R*(ω) = χ^(3)^(ω). For normal incidence on the (101) crystal surface, the orthorhombic space group *Pnma* allows Kerr signals from $\chi_{xxxx}^{\left(3\right)},\chi_{yyyy}^{\left(3\right)},\chi_{xxyy}^{\left(3\right)}=\chi_{xyyx}^{\left(3\right)}=\chi_{xyxy}^{\left(3\right)}$ and $\chi_{yyxx}^{\left(3\right)}=\chi_{yxxy}^{\left(3\right)}=\chi_{yxyx}^{\left(3\right)}$ (*35*), while the cubic space group *Pm3m* allows for $\chi_{xxxx}^{\left(3\right)}=\chi_{yyyy}^{\left(3\right)}$ and $\chi_{xxyy}^{\left(3\right)}=\chi_{xyyx}^{\left(3\right)}=\chi_{xyxy}^{\left(3\right)}=\chi_{yyxx}^{\left(3\right)}=\chi_{yxxy}^{\left(3\right)}=\chi_{yxyx}^{\left(3\right)}$. The *Pnma* space group applies to CsPbBr_3_ in its orthorhombic phase, which is the case for all temperatures considered in this work. The *Pm3m* space applies to MAPbBr_3_ for its room temperature cubic phase. All allowed tensor elements were assumed to have the same magnitude.

Simulations for an electronic response only and without optical anisotropy are shown for various thicknesses in fig. S16. This applies to MAPbBr_3_ single crystals at room temperature when this material is in the cubic phase. Simulations for an electronic response only and with optical anisotropy are shown for various thicknesses in fig. S17. This applies to MAPbBr_3_ in its low-temperature orthorhombic phase and CsPbBr_3_, which is orthorhombic for all temperatures considered in this work. The effect of optical anisotropy on the TKE is very dependent on the azimuthal angle of the crystal. Results are shown for two different azimuthal angles: 0° and 45° angle between crystal axis and probe polarization in fig. S17.

Figure S17 shows that the oscillatory features due to propagation effects and static birefringence cannot explain the oscillations observed in low-temperature MAPbBr_3_ single crystals and thin films. To simulate this oscillatory signal, we have to consider both electronic and phonon contributions to *R* = *R*_e_ + *R*_ph_ alongside static birefringence. The chosen simulation parameters are given in the main text and were chosen in accordance with the Lorentzian fits to our experimental data in fig. S10. The instantaneous contribution to *R* is *R*_e,0_/*R*_ph,0_ times larger than the phononic contribution when the respective spectral amplitude of the responses is integrated in the 0- to 10-THz range.

## Supplementary Materials

### This PDF file includes:

Sections S1 to S5 Figs. S1 to S17
