# Supplementary material for: Nonlinear terahertz control of the lead halide perovskite lattice
Source: Sci Adv. 2023 May 24;9(21):eadg3856. doi: 10.1126/sciadv.adg3856 (PMC10208573; doi:10.1126/sciadv.adg3856)
Supplement: Supplementary file 1 — Sections S1 to S5 Figs. S1 to S17 [file sciadv.adg3856_sm.pdf]

Science Advances
AAAS

Supplementary Materials for

Nonlinear terahertz control of the lead halide perovskite lattice

Maximilian Frenzel *et al.*

Corresponding author: Sebastian F. Maehrlein, maehrlein@fhi.mpg.de

Sci. Adv. **9**, eadg3856 (2023)
DOI: 10.1126/sciadv.adg3856

This PDF file includes:

Sections S1 to S5
Figs. S1 to S17

Supplementary information

Section S1: $CsPbBr_3$ TKE temperature dependence

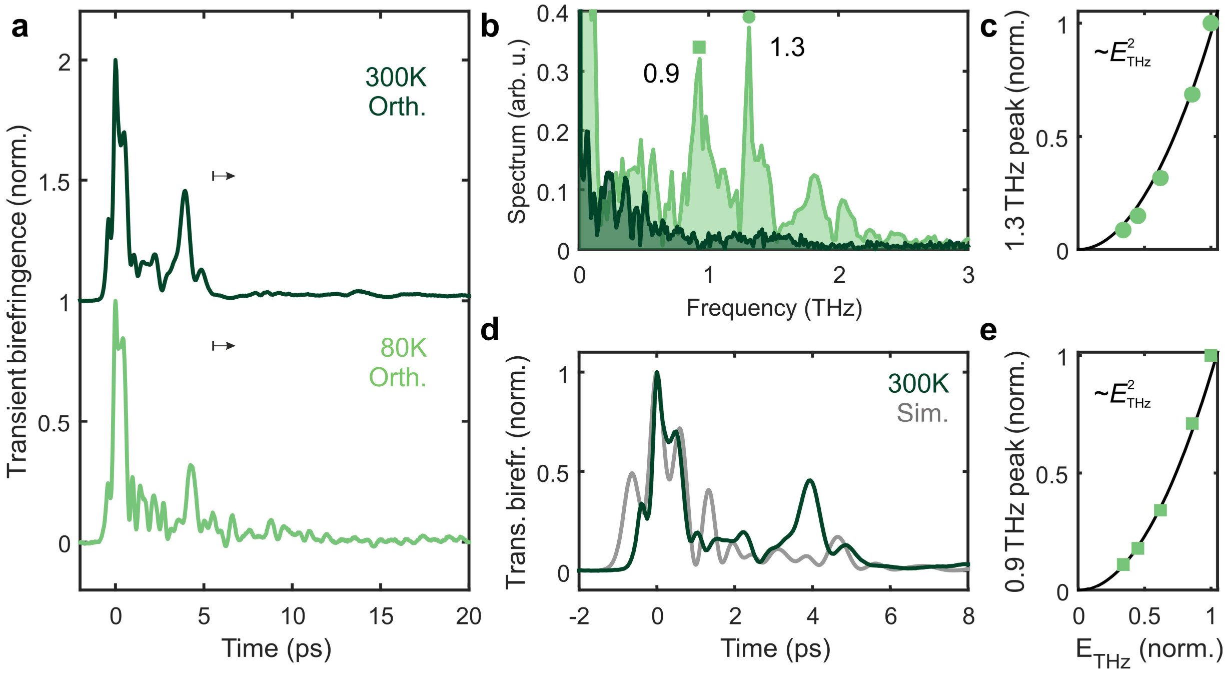

Fig. S1 | TKE temperature evolution in $CsPbBr_3$. a. TKE in $CsPbBr_3$ at RT and 80K. In contrast to $MAPbBr_3$, where the structural phase changes for lower temperatures (from cubic to tetragonal to orthorhombic), $CsPbBr_3$ remains in the orthorhombic phase as the temperature is lowered. This is also reflected in the overall TKE shape. However, additional oscillations are visible on the longer timescales at 80K. The birefringent orthorhombic phase at room temperature leads to oscillatory propagation effects in the region $0 < t < 5$ ps (see simulations in Fig. S17). **b.** Fourier transforming the oscillations of a. for $t > 5$ ps (black arrow) reveals two main frequency components of 0.9 and 1.3 THz, as well as two weaker components around 2 THz. These frequencies agree well with the phonon modes in the static Raman spectra of $CsPbBr_3$ (*46*). **c, e.** THz fluence dependence reveals that both oscillation amplitudes (for 0.9 and 1.3 THz) scale quadratically with the THz electric field. **d.** Comparison between simulation for an anisotropic material (100 μm thick and 22.5° azimuthal angle between crystal axis and probe polarization) considering an electronic response only and experimental room temperature $CsPbBr_3$ TKE. This shows that the complex $CsPbBr_3$ TKE signal may be understood in terms of an instantaneous electronic polarization response alongside anisotropic light propagation.

Section S2: Estimating the THz nonlinear refractive index of $MAPbBr_3$

Fig. S7 shows a comparison between the TKE in $MAPbBr_3$ single crystal and Diamond. The measured TKE signal strength $S(d) = \Delta I/I_0$, where I_0 is the total probe intensity measured by the photodiodes and ΔI is the intensity difference, is proportional to $\Delta n \omega_{pr} d/c_0$ in Diamond, where d is the sample thickness and ω_{pr} is the probing frequency.
This simple relation holds because there is no significant THz dispersion in Diamond. However, due to significant THz absorption and dispersion, this relation does not hold in $MAPbBr_3$ as seen in Fig. S6b. For $MAPbBr_3$, $S(d)$ may rather be approximated by $\frac{\Delta n \omega_{pr}}{c_0} f(d)$, where

$$f(d) = \int_0^d dz \int_0^\infty d\omega E_{THz}^2(\omega) \exp(-\alpha(\omega) z) \,/\, \int_0^\infty d\omega E_{THz}^2(\omega). \tag{S1}$$

Here, $E_{THz}(\omega)$ is the THz pump spectrum and α is the absorption of $MAPbBr_3$ as extracted from the complex refractive index data in Fig. S14.
Since $\Delta n = n_2 c_0 \epsilon_0 E_{THz}^2$, we may estimate n_2 of $MAPbBr_3$ using:

$$n_2^{MA} = \frac{S_{MA}(d_{MA}) d_D}{S_D(d_D) f(d_{MA})} n_2^D. \tag{S2}$$

n_2^D of Diamond has been measured to be 3×10^{-16} cm²/W for 1 THz pump and 800 nm optical probing (*44*). Based on $\frac{S_{MA}}{S_D} = 9.4$, $f(d_{MA} = 500\ \mu m) = 47 \mu m$, we therefore estimate n_2^{MA} to be 2×10^{-14} cm²/W, roughly 80 times higher than n_2^D for 1 THz pump and 800 nm optical probing. For comparison, n_2^{MA} has been previously measured in the near-infrared spectral region using the Z-scan technique (*43*). They found a similar order of magnitude of $n_2^{MA} = 9.5 \times 10^{-14}$ cm²/W at 1000 nm wavelength.

Section S3: Estimation of the THz-induced twist angle modulation

We can estimate an order of magnitude for the THz-induced twist angle change by comparison with the experiment by Kim et al (*24*). Kim et al. probed the transient white light absorption of $MAPbI_3$, broadly covering $MAPbI_3$'s electronic bandgap, under similar THz excitation conditions as in this work. They reported a modulation of the bandgap by $\Delta E_g \approx 0.3$ meV using THz excitation fields of 100 kV/cm, likely also driven by the 1 THz twist mode of $MAPbI_3$. Extrapolating this result to our experimental conditions with excitation fields exceeding 1 MV/cm and assuming a linear scaling with the phonon amplitude $\Delta E_g \propto Q \propto E_{THz}^2$, we may roughly estimate the transient modulations of the bandgap to be on the order of 30 meV. Based on studies of the static structural modification by chemical engineering, this bandgap-change corresponds to an octahedral twist of about 1.5° as shown by M.R. Filip et al. (*17*).

Section S4: Non-oscillatory background removal

The incoherent background predominantly stems from the electronic polarizability in the $MAPbBr_3$ single crystal (Fig. 3A). There, it is produced by a walk-off between pump and probe, and varying penetration depths across the broad THz spectrum (see Fig. S14). We remove the

incoherent background by convoluting the measured THz electric field $E_{\mathrm{THz}}^2(t)$ with a $\delta(t)$-function (local instantaneous electronic polarization response) and a double exponential decay (due non-local propagation effects):

$$S_{\mathrm{BG}}(t) = E_{\mathrm{THz}}^2(t) * \left(\delta(t) + \left(\frac{R_1}{\tau_1}\right)\exp\left(-\frac{t}{\tau_1}\right) + \left(\frac{R_2}{\tau_2}\right)\exp\left(-\frac{t}{\tau_2}\right)\right) \tag{S3}$$

Due to the dominance of propagation effects we only use the fitted functions in Fig. S2 to remove the incoherent background.

For the thin film sample (Fig. 3B), where propagation effects are negligible, the incoherent tail nearly vanishes, supporting the non-local origin of the single crystal tail. We also fitted the weak remaining incoherent background of the thin film TKE data with the above convolution function in Fig. S2b. However, the caveat of the thin film data is the small non-oscillatory TKE contribution from substrate (see Figs. S11 and S12). We therefore refrain from interpreting the incoherent background dynamics here too.

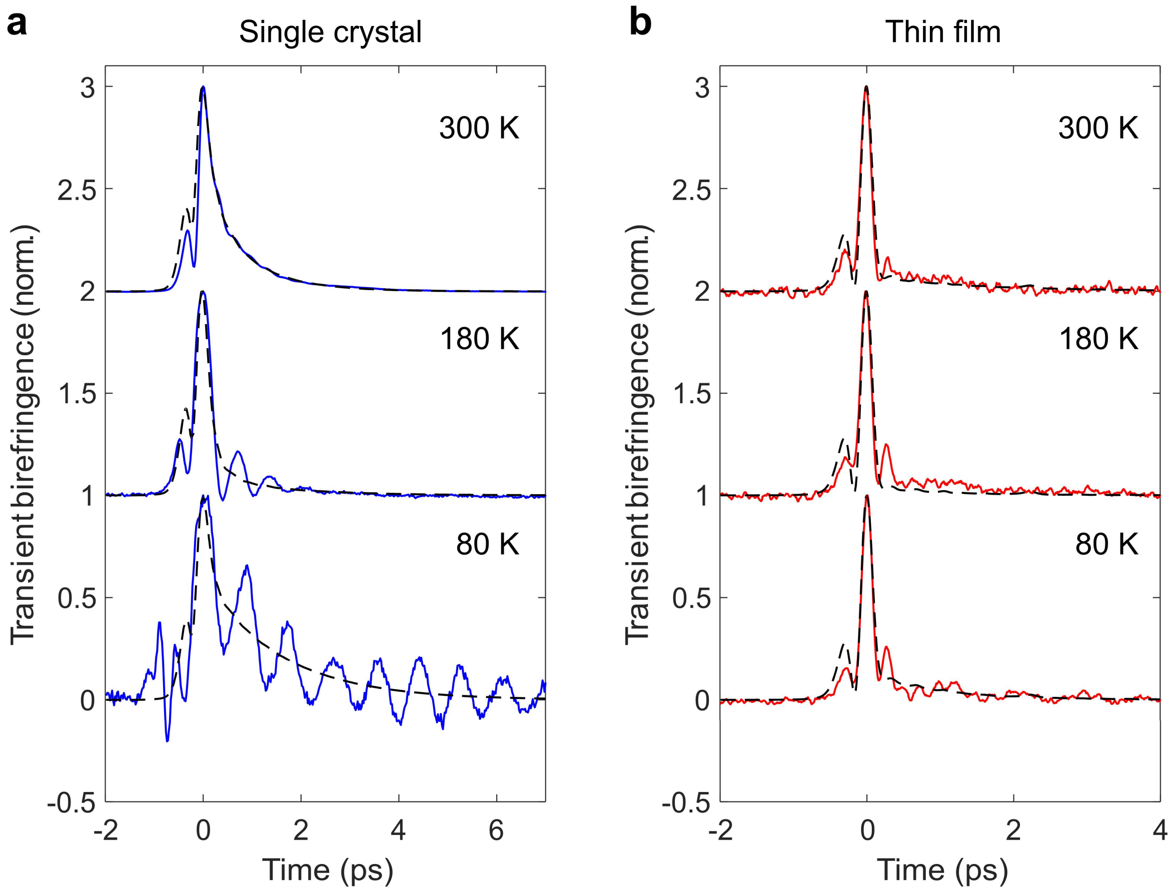

Fig. S2 | Non-oscillatory TKE dynamics of single crystal and thin film $MAPbBr_3$. $MAPbBr_3$ **a.** single crystal and **b.** thin film TKE for 300K, 180K, and 80K with corresponding fits to the tail dynamics using a convolution of E_{THz}^2 with a temporal delta function and bi-exponential decay.

Section S5: Toy model - Instantaneous TKE response

At lower temperatures, stronger THz absorption decreases the THz penetration depth (*53*). Therefore, the interaction volume of the THz pump and the probe field decreases in the bulk samples, leading to a reduced amplitude of the TKE peak around $t = 0$ ps. We can describe this effect in a toy model with the local instantaneous TKE response being proportional to $E_{\mathrm{THz}}^2(z, t)$, and introducing pump probe walk-off and absorption similar to (*44*):

$$S_{\mathrm{TKE}}(t) = \int_0^L E_{\mathrm{THz}}^2(z = 0,\ t + \beta z) \exp(-\alpha_{\mathrm{THz}} z)\, \mathrm{d}z \tag{S4}$$

where the inverse velocity mismatch $\beta = (n_{\mathrm{probe}} - n_{\mathrm{THz}})/c$ accounts for walk-off effects, leading to "smearing out" of the instantaneous E_{THz}^2 feature. The absorption coefficient α_{THz} determines the penetration depth and thus the effective interaction volume. Both walk-off and absorption give rise to a non-oscillatory background in the form of an exponential decay.

Higher THz absorption at lower temperatures also leads to a reduced interaction volume and thus lower signal S_{TKE}. The thin film however is much thinner than the penetration depth $1/\alpha_{\mathrm{THz}}$ and thus its TKE signal is not affected by changes in the absorption coefficient. We simulate these effects based on the above equation for the $MAPbBr_3$ single crystal of 500 µm (Fig. S3a) and thin film of 0.4 µm thickness (Fig. S3b). For this simulation, we used a THz refractive index of 7, which is the refractive index of $MAPbBr_3$ at around 1 THz in Fig. S14. Similarly, the chosen absorption coefficient values are roughly in the range of the absorption coefficient values of $MAPbBr_3$ at around 1 THz.

In contrast to the Four-wave-mixing simulation shown in the main paper, this toy model does not capture the material's dispersion and static birefringence and should therefore not be used as a quantitative estimate. Nonetheless, the toy model qualitatively depicts, why the instantaneous $t = 0$ ps feature is roughly independent of temperature in the thin film, but nearly vanishes in comparison to the oscillatory response in the bulk samples at 80 K.

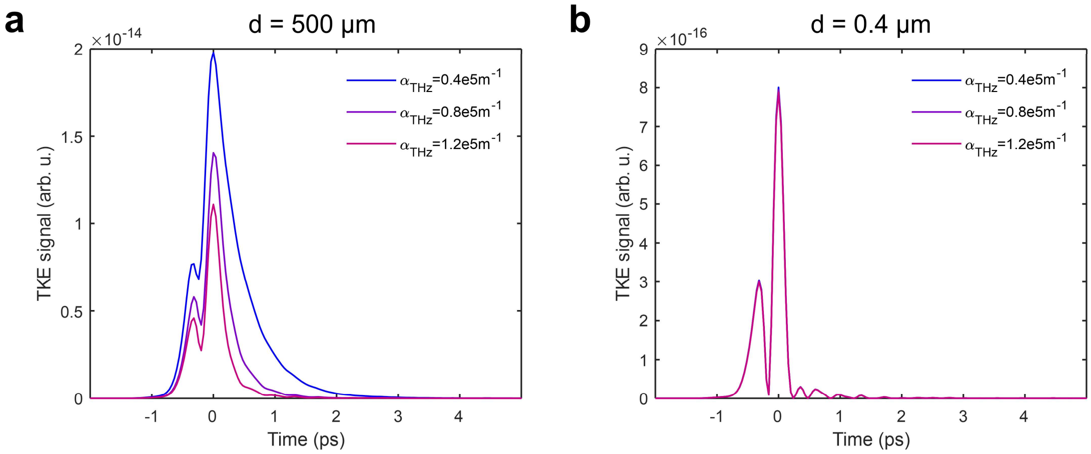

Fig. S3 | Toy model simulation to illustrate effect of THz absorption on instantaneous TKE response. The simulation is carried out for three different THz absorptions and for two different sample thicknesses. **a.** simulation for 500 μm, corresponding to the expected trend in $MAPbBr_3$ single crystals, where higher THz absorption at lower temperatures leads to a reduced $t = 0$ ps peak and different incoherent background. **b.** simulation for 0.4 μm, corresponding to the $MAPbBr_3$ thin film, where the TKE does not change and is therefore less sensitive to changes in THz absorption.

Supplementary figures

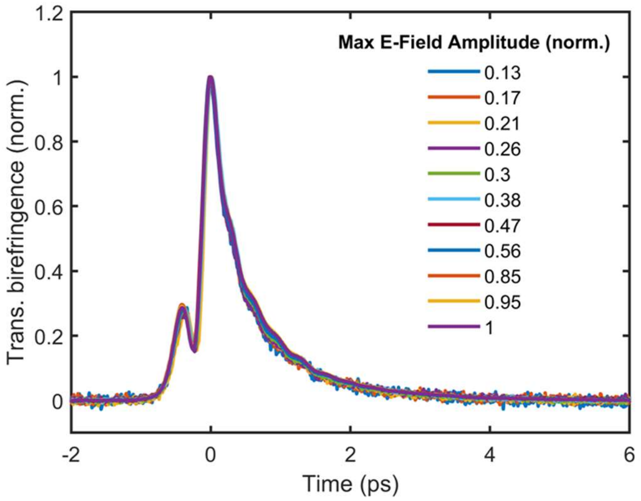

Fig. S4 | $MAPbBr_3$ TKE temporal dependence on THz fluence. Normalized experimental TKE of $MAPbBr_3$ single crystal at room temperature for various THz fluences showing that the temporal evolution is not affected by the THz-field strength. Fig. 2 in the main text already showed that the $t = 0$ ps peak scales quadratically with the THz field amplitude.

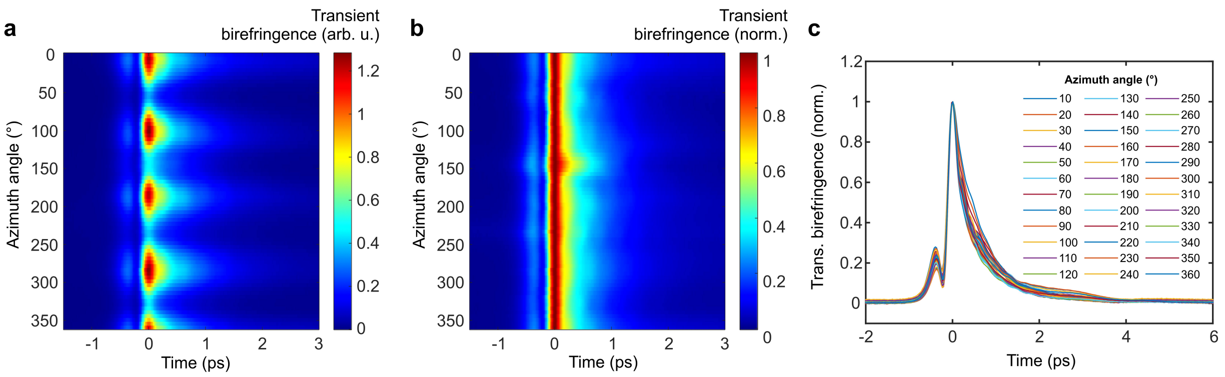

Fig. S5 | $MAPbBr_3$ TKE azimuthal angle dependence at room temperature. a. TKE signal showing the 4-fold rotational symmetry of the measured signal. **b, c.** TKE signal is normalized to show that the time constant of the tail is independent of azimuthal angle. This agrees with the simulations for an isotropic material in Fig. S16, where the origin of the exponential tail is high absorption, dispersion and pump-probe walk-off, which do not depend on the crystal azimuthal angle. Note that the azimuthal angle is not calibrated with respect to the crystal axes in this figure.

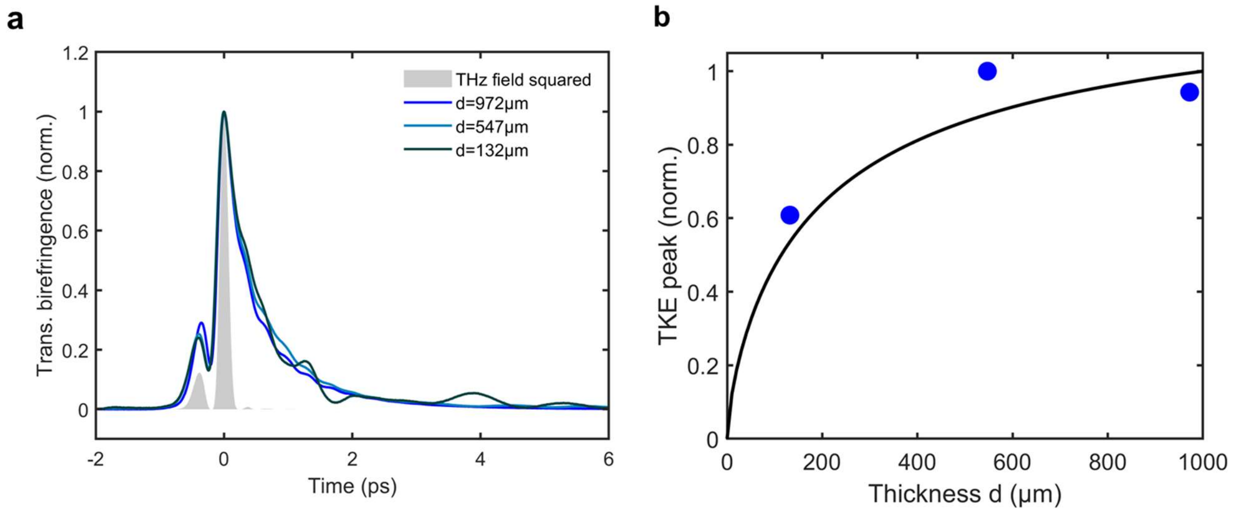

Fig. S6 | $MAPbBr_3$ TKE dependence on sample thickness. a. Normalized experimental TKE thickness dependence of $MAPbBr_3$ at room temperature. The results agree well with the simulations in Fig. S16. **b.** shows the measured TKE peak signal as a function of sample thickness. The black line shows the expected signal dependence when accounting for strong THz absorption and dispersion using the formula $S(d) = \int_0^d dz \int_0^\infty d\omega E_{\mathrm{THz}}^2(\omega)\exp(-\alpha(\omega)z) / \int_0^{1000} dz \int_0^\infty d\omega E_{\mathrm{THz}}^2(\omega)\exp(-\alpha(\omega)z)$, where $E_{\mathrm{THz}}(\omega)$ is the THz pump spectrum and α is the absorption of $MAPbBr_3$ as extracted from the complex refractive index data in Fig. S14.

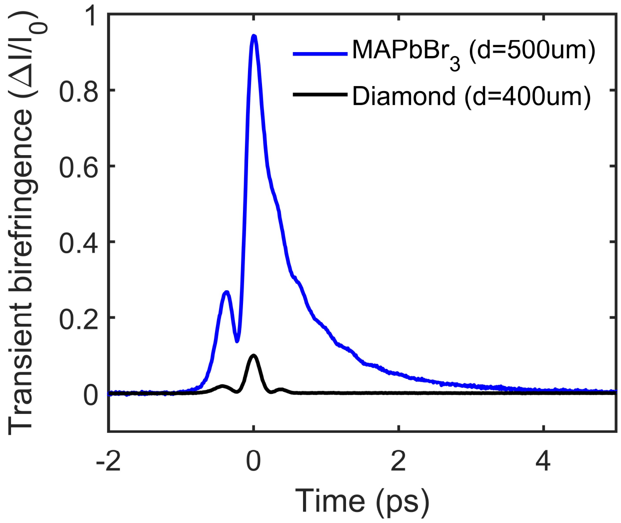

Fig. S7 | Comparison between TKE in $MAPbBr_3$ and Diamond for estimating the THz nonlinear refractive index n_2. Diamond has already been shown to have a strong THz-induced Kerr nonlinearity and be a good nonlinear material in the THz range (*45*). n_2 of Diamond has been measured to be 3×10^{-16} cm^2/W for 1 THz pump and 800 nm optical probing (*44*). For a 500 μm thick $MAPbBr_3$ single crystal, the TKE peak signal is about 10 times bigger than for a 400 μm thick Diamond.

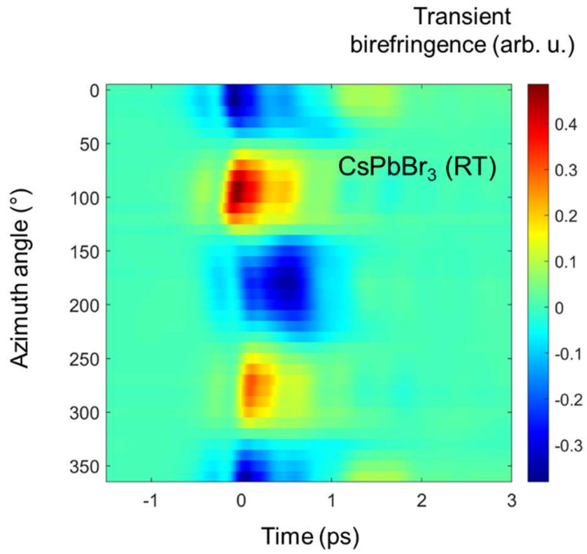

Fig. S8 | $CsPbBr_3$ TKE azimuthal angle dependence at room temperature. Although the main peak exhibits a 4-fold symmetry, the temporal evolution as a function of azimuthal angle is more complex than for $MAPbBr_3$. As $CsPbBr_3$ is in the orthorhombic phase at room temperature, this extra complexity might be explained by additional static birefringence and resulting anisotropic light propagation as can be seen in Fig. S17. Note that the azimuthal angle is not calibrated with respect to the crystal axes in this figure.

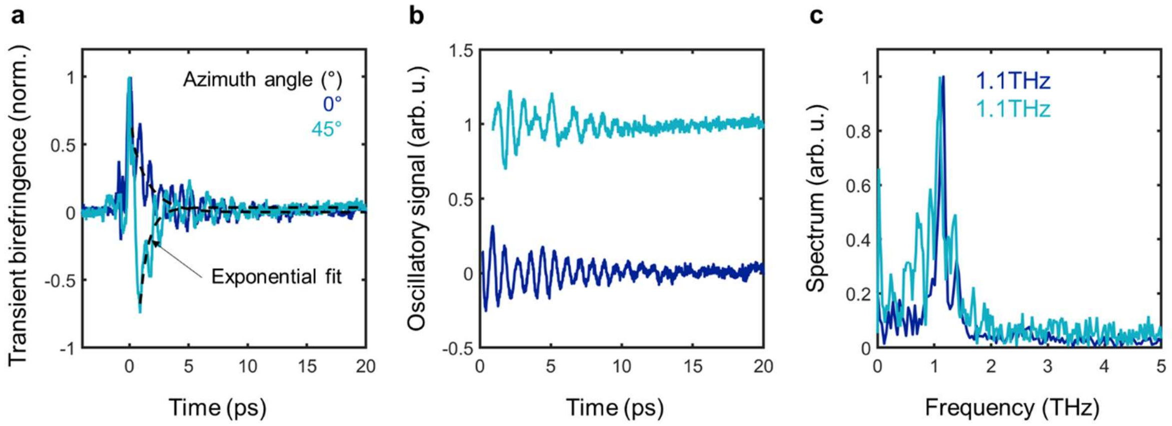

Fig. S9 | $MAPbBr_3$ single crystal TKE at 45° azimuthal angle at 80K. a. $MAPbBr_3$ TKE at 80K for 0° and about 45° azimuthal angle. $MAPbBr_3$ is orthorhombic at 80K, which might explain the different overall signal shape for both orientations. However, in both TKEs we can see a strong oscillatory signal. **b.** By subtracting off fits to the tails (dotted line in a.) for the TKEs at 0° and 45°, we extract the oscillatory signals. **c.** Fourier transforming the oscillatory signals in b. reveals that the same 1.1 THz mode dominates the oscillatory response at 0° and 45° azimuthal angle.

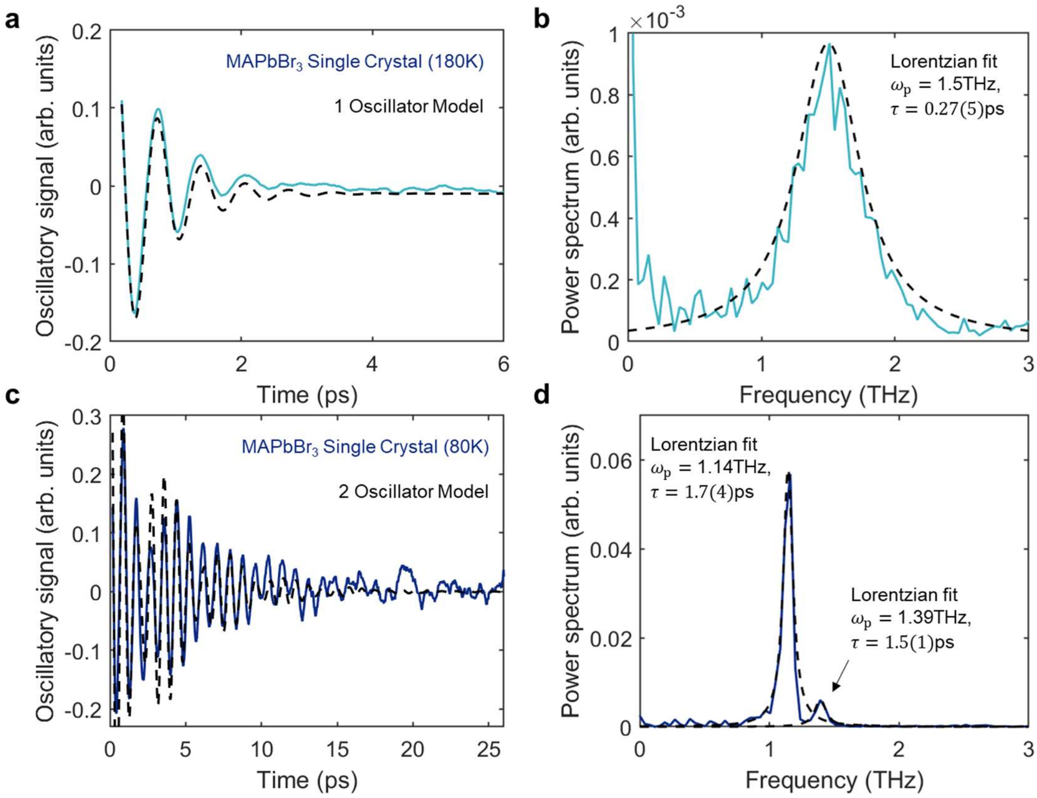

Fig. S10 | Lorentzian fits to spectral peaks in $MAPbBr_3$ single crystal at 180K and 80K. a,c., Oscillatory signals extracted from the $MAPbBr_3$ TKE at 180K and 80K in Fig. 3A respectively. The signals are extracted by subtracting off exponential fits to the tails from the main TKE signals. **b.** The modulus squared of the Fourier transform of the oscillatory signal at 180K shows a broad peak at 1.5 THz, which we fit with a Lorentzian. The FWHM of the Lorentzian amplitude is $\Delta\nu_{\mathrm{FWHM}}$ =0.58 THz. This corresponds to a phonon lifetime of $\tau = 1/(2\pi\Delta\nu_{\mathrm{FWHM}}) = 0.27$ ps. **d.** The modulus squared of the Fourier transform of the oscillatory signal at 80K shows two peaks at 1.14 THz and 1.39 THz. By fitting Lorentzians, we obtain phonon lifetimes of 1.7(4) ps and 1.5(1) ps for the two peaks respectively.

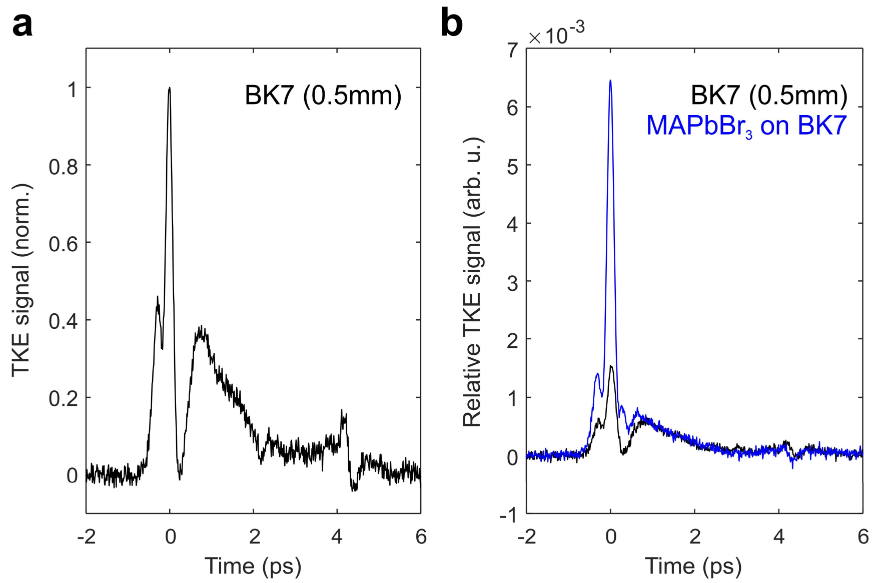

Fig. S11 | BK7 TKE at room temperature. a. TKE of a BK7 substrate with 0.5 mm thickness. **b.** shows the measured BK7 TKE relative to the TKE of the $MAPbBr_3$ thin film, which is on top of a BK7 substrate with 0.5 mm thickness. The BK7 substrate contribution to the instantaneous $t = 0$ ps TKE of the $MAPbBr_3$ thin-film is small. However, there is a significant non-oscillatory contribution between 0.5 and 4 ps, which explains the small tail in the $MAPbBr_3$ thin-film traces.

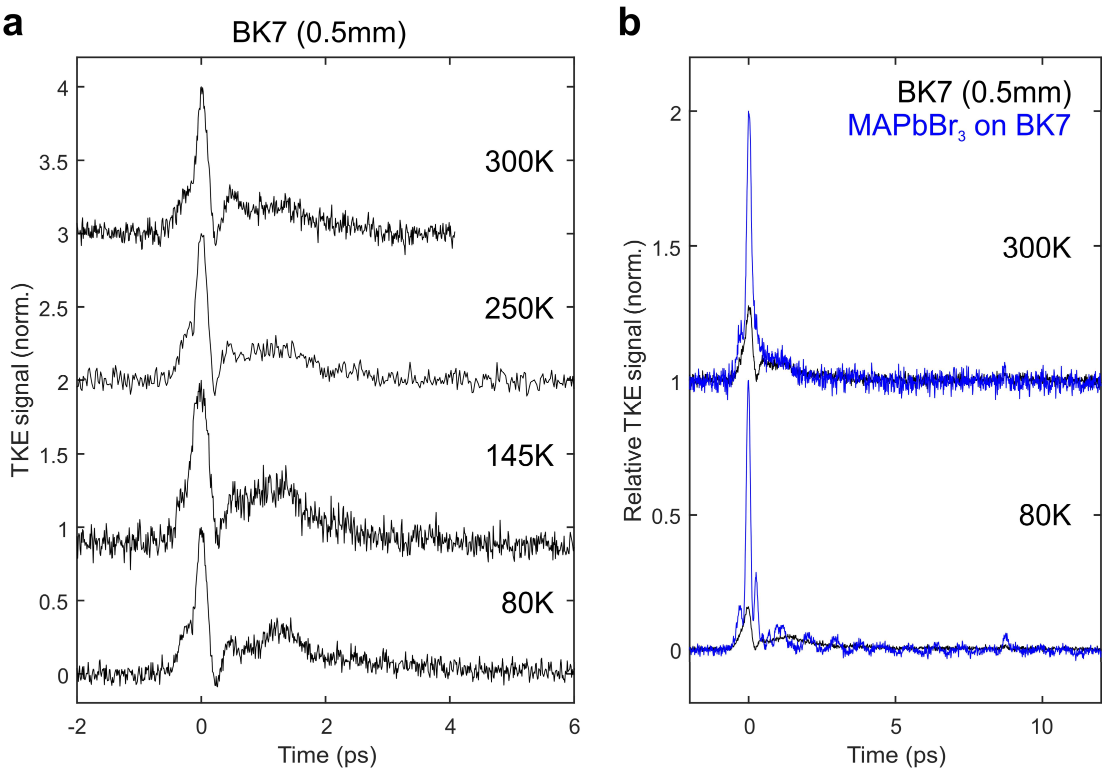

Fig. S12 | BK7 TKE for various temperatures. a. TKE temperature dependence of BK7 substrate with 0.5 mm thickness. **b.** shows the relative TKE signal of the BK7 substrate compared to $MAPbBr_3$ thin film on top of a BK7 substrate for room temperature and 80K. The shown $MAPbBr_3$ thin film TKE were normalized and offset for clarity for both temperatures. It can be seen that the BK7 substrate contribution to the $MAPbBr_3$ thin film TKE remains the same for all temperatures considered in this work. Its contribution to the main $t = 0$ ps peak in the $MAPbBr_3$ thin film remains small, but it has a non-oscillatory contribution between 0.5 and 4 ps, which looks like a small exponential tail.

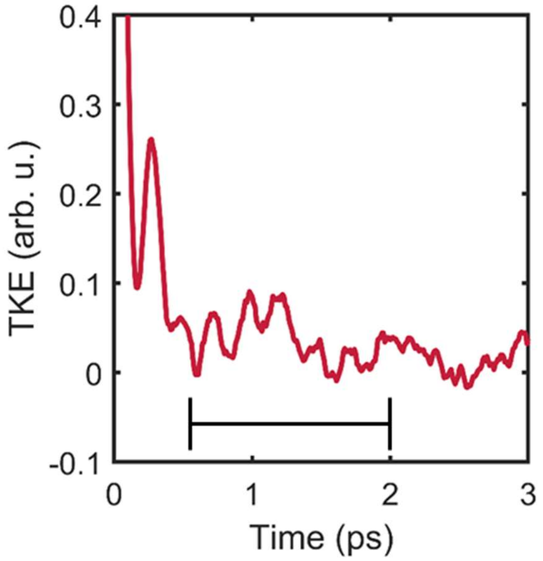

Fig. S13 | Short time $MAPbBr_3$ thin film TKE 4.2 THz oscillations at 80K. The broader 4.2 THz thin film feature in Fig. 3D corresponds to small wiggles in the short time window of $0.5 < t < 2$ ps (indicated by the black bar), which end abruptly. This feature might be related to interference effects between the substrate and the $MAPbBr_3$ thin film.

Simulation figures

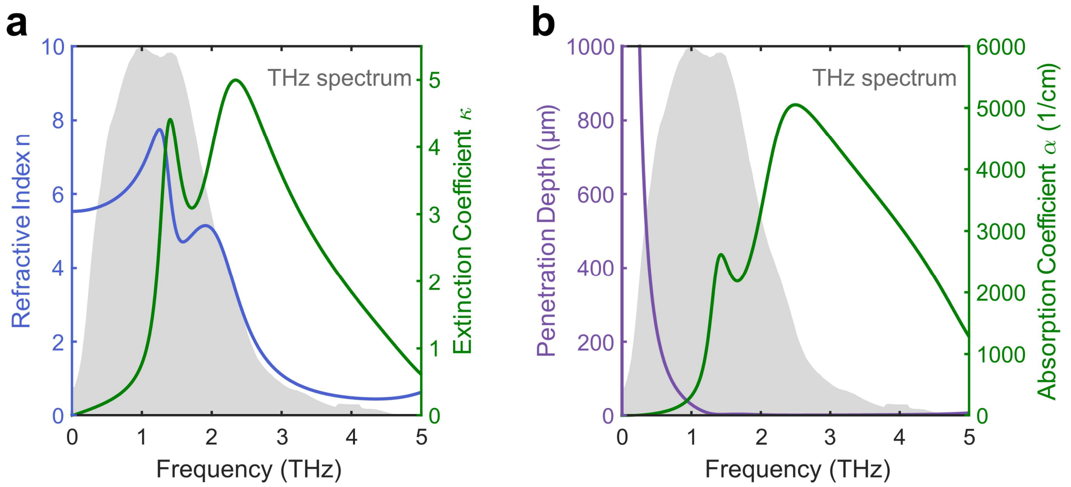

Fig. S14 | Dispersion of $MAPbBr_3$ in the THz region. a. Refractive index n and extinction coefficient κ of $MAPbBr_3$ calculated using the dielectric function from Sendner et al. (*6*). **b.** Absorption coefficient is calculated using relation $\alpha = 4\pi\kappa/\lambda$. The penetration depth is equal to $1/\alpha$.

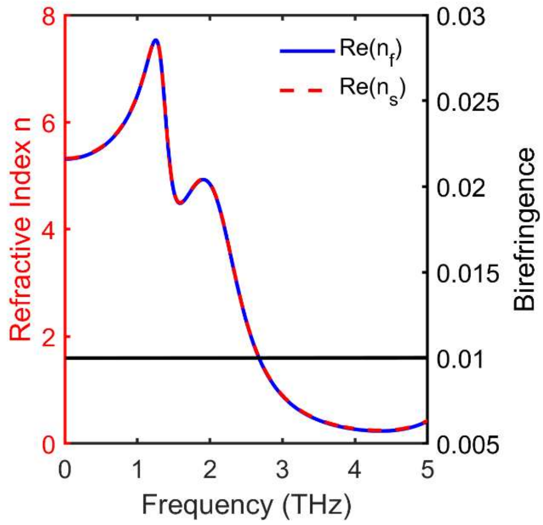

Fig. S15 | Extrapolated static birefringence of $MAPbBr_3$ for the simulations of the low temperature orthorhombic phase. In the optical region, the refractive index of $CsPbBr_3$ is used as measured using the 2D-OKE (*39*). The static birefringence of $CsPbBr_3$ is then extrapolated to the THz region. n_f and n_s correspond to the refractive index along the fast and slow crystal axes and static birefringence is defined as the difference between n_f and n_s.

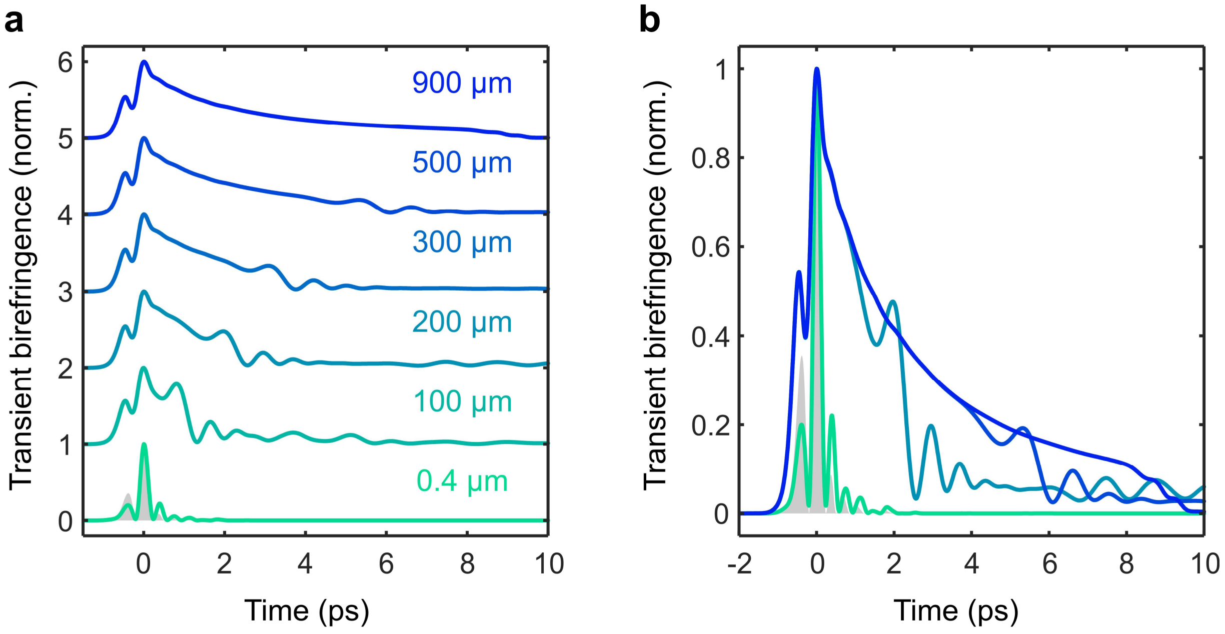

Fig. S16 | Four-wave-mixing simulation for isotropic $MAPbBr_3$ for various thicknesses assuming an instantaneous electronic hyperpolarizability response only. a. shows the normalized TKE signal for various thicknesses. For thicknesses larger than 100 µm, we can see an exponential tail with a decay time constant largely independent of thickness. **b**. The normalized TKE signals for various thicknesses are plotted on top of each other. On top of the exponential tail, there are small modulations, whose onset depends on the thickness. The onset time can be roughly estimated by the t_1 time ($t_1 = (n_{g,f}(\omega_{\mathrm{THz}}) - n_{g,f}(\omega_{\mathrm{pr}}))d/c_0$), where d is the sample thickness and n_g is the group velocity refractive index (*39*).

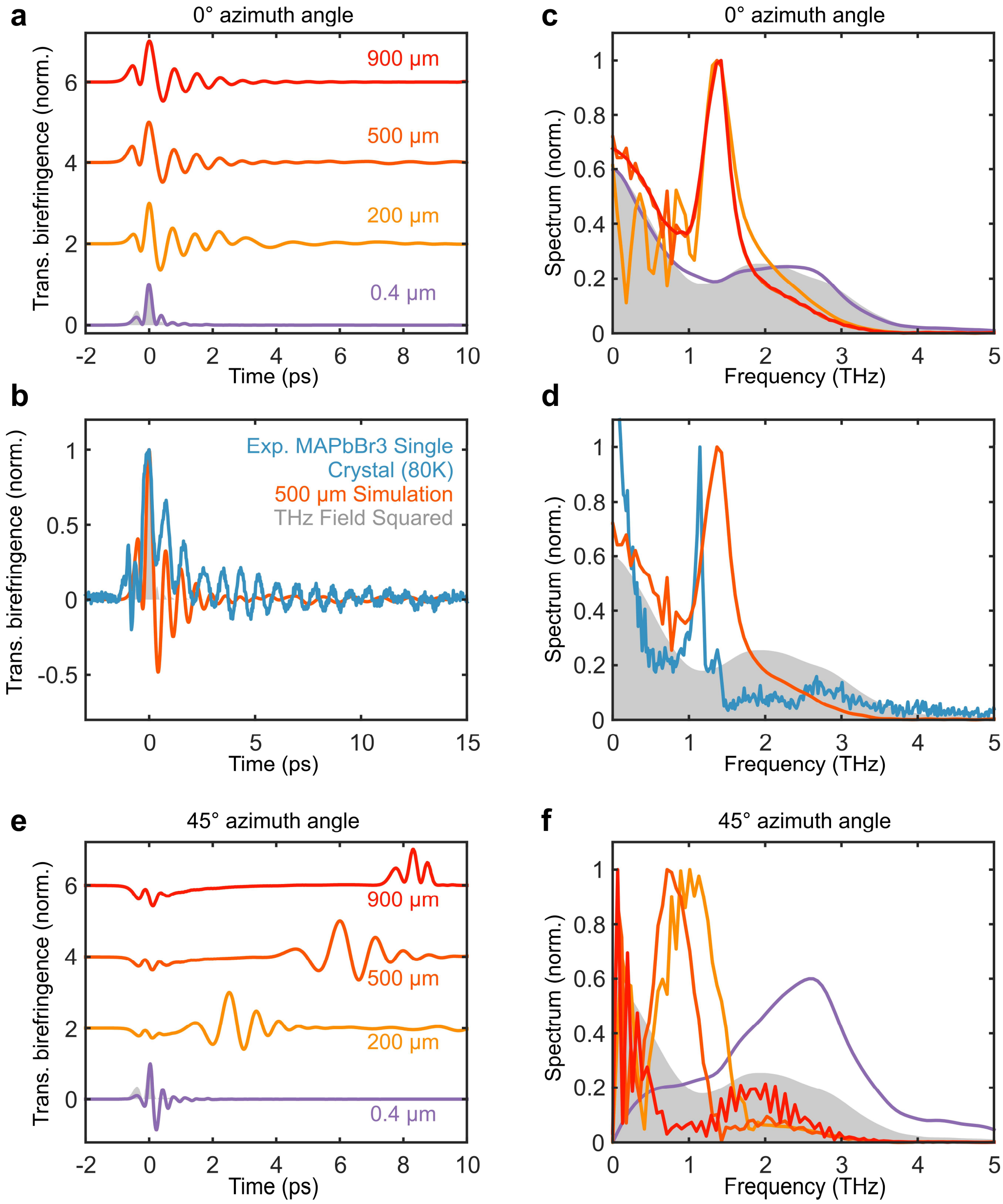

Fig. S17 | Four-wave-mixing simulation for birefringent $MAPbBr_3$ assuming an instantaneous electronic hyperpolarizability response only. In contrast to the isotropic case simulation in Fig. S16, the azimuthal angle of the model crystal matters for the temporal TKE shape. **a-d**. Results for 0° azimuthal angle of crystal with respect to probe pulse polarization are shown for various thicknesses. For this angle, the birefringence experienced by the probe is maximized. For 0° azimuthal angle and for all thicknesses larger than 200 µm, we can see the appearance of a short-lived oscillatory signal of around 1.4 THz in a-d. The simulated signal seems independent of sample thickness for thicknesses between 200 and 900 µm. The oscillations in the simulated signals arise due to static birefringence, and their lifetime is roughly given by the time required for the probe and THz pulse to walk-off. As shown in b., and d., these oscillations would be too short-lived to explain the observed oscillations in the $MAPbBr_3$ single crystal at 80K. On

the other hand, the oscillations in $CsPbBr_3$ in Fig. S1a before $t = 5$ ps are very likely caused by static birefringence. For a small thickness, such as 0.4 μm, these oscillations due to static birefringence disappear. Since we observe oscillations of the same frequency in the $MAPbBr_3$ single crystals and thin films, the simulations therefore confirm that the observed $MAPbBr_3$ oscillations at 80K are caused by a phonon and not by static birefringence. The absence of oscillatory propagation effects in $MAPbBr_3$ at 80K might be explained by the nearly vanishing instantaneous hyperpolarizability contribution (Fig. 3A (lowest trace) or blue trace in b.) likely due to a smaller THz penetration depth. **e-f.** Results for 45° azimuthal angle for various thicknesses. For this angle, the birefringence experienced by the pump is maximized. The peak $t = 0$ ps is diminishingly small in comparison to the oscillatory features that happen at later times, which is due to the input tensor symmetry of R. The small oscillatory features correspond to internal reflections - similar to the small modulations on top of the tail in Fig. S16. The onset time for these oscillatory features can be roughly estimated by the t_1 time.
